# Infection with COVID-19 Complicated by Sinus Arrest

**DOI:** 10.1155/2024/5361758

**Published:** 2024-05-16

**Authors:** Guojun Zhang, Shuai He, Lu Lin, Pengcheng Gan

**Affiliations:** ^1^Department of Infectious Disease, The First People's Hospital of Fuyang Hangzhou, Fuyang District, Hangzhou, Zhejiang, China; ^2^Department of Hand and Foot Surgery, Taizhou Hospital of Zhejiang Province Affiliated to Wenzhou Medical University, Linhai District, Taizhou, Zhejiang, China; ^3^Department of Intensive Care Rehabilitation, Taizhou Hospital of Zhejiang Province Affiliated to Wenzhou Medical University, Luqiao District, Taizhou, Zhejiang, China

## Abstract

As a respiratory tract-transmitted disease, coronavirus disease 2019 (COVID-19) exerts a profound immune injury effect, leading not only to pulmonary impairment but also to cardiac complications. We present a case of a 79-year-old woman, who had previously contracted COVID-19 and subsequently developed sinus arrest (SA) following her second infection. The longest asystole time detected by Holter monitoring was 7.2 seconds. Although the patient met criteria for permanent pacemaker implantation, her family declined this intervention and conservative management was pursued instead. However, after a period of observation, the patient's SA resolved. The present case study describes a patient who experienced SA upon reinfection with COVID-19, which was not present during the initial infection. It emphasizes the impact of COVID-19 on cardiac health, particularly its potential to induce arrhythmias. In addition, it is worth noting that the arrhythmia induced by a COVID-19 infection may show reversibility, suggesting that a permanent pacemaker might not be the priority option if further pacing therapy is being considered.

## 1. Introduction

In recent years, the global coronavirus disease 2019 (COVID-19) pandemic has drawn increasing attention to its detrimental effects. In addition to respiratory damage, severe acute respiratory syndrome coronavirus 2 (SARS-CoV-2) can also impact the central nervous, cardiovascular, and digestive systems [1]. Sinus arrest (SA), also known as sinus pause, is an arrhythmia type associated with dysfunction of the sinus node. It can be triggered by various factors such as medication usage, inflammation, ischemia, and injury [2]. The occurrence of SA may result in symptoms such as dizziness, dyspnea, syncope, and other manifestations [3]. Furthermore, it can give rise to circulatory ischemia, a life-threatening condition [3]. The current evidence suggests that SA can be secondary to COVID-19 infection; for example, the study conducted by Ansari et al. [4] reviewed 30 case reports/case series involving 67 patients with bradyarrhythmias in COVID-19 infection, revealing that SA was observed in 17.9% of the patients.

There are a lot of data showing that COVID-19 is highly transmissible and individuals who have recovered may be susceptible to reinfection [5]. Additionally, reinfection gives rise to additional health risks beyond the initial infection, encompassing sequelae across a diverse range of organ systems [6]. We present a case where reversible SA occurred following the second episode of COVID-19 infection but not during the initial infection.

## 2. Case Presentation

The patient, a 79-year-old woman with a medical history of hypertension, type 2 diabetes mellitus, paroxysmal atrial fibrillation, and cerebral infarction, was admitted to our hospital for long-term rehabilitation due to tetraplegia and impaired consciousness resulting from the cerebral infarction. This patient experienced two episodes of novel coronavirus pneumonia during hospitalization. During her first COVID-19 infection, she developed respiratory failure and received invasive mechanical ventilation. Given the patient's lack of adequate indications for decannulation, a tracheotomy procedure was performed. Following recovery from COVID-19, the ventilator was gradually withdrawn and a tracheostomy tube was permanently indwelled. Five months later, the patient presented with fever and respiratory failure again and tested positive for SARS-CoV-2 nucleic acid in sputum samples. We considered that the patient had a second infection with COVID-19. At the onset of the infection, the patient exhibited a temperature ranging from 37 to 39°C and maintained sinus rhythm with intermittent episodes of atrial fibrillation. Her heart rate ranged between 60 and 100 beats per minute, and her blood pressure remained within normal limits. She also presented with a troponin-I level of 0.063 ng/mL (0.0–0.03 ng/mL), and an echocardiogram revealed mildly impaired left ventricular diastolic function, with a left ventricular ejection fraction of 62% and mild aortic regurgitation. Liver and kidney functions as well as electrolyte levels were within normal range.

**Figure 1:**
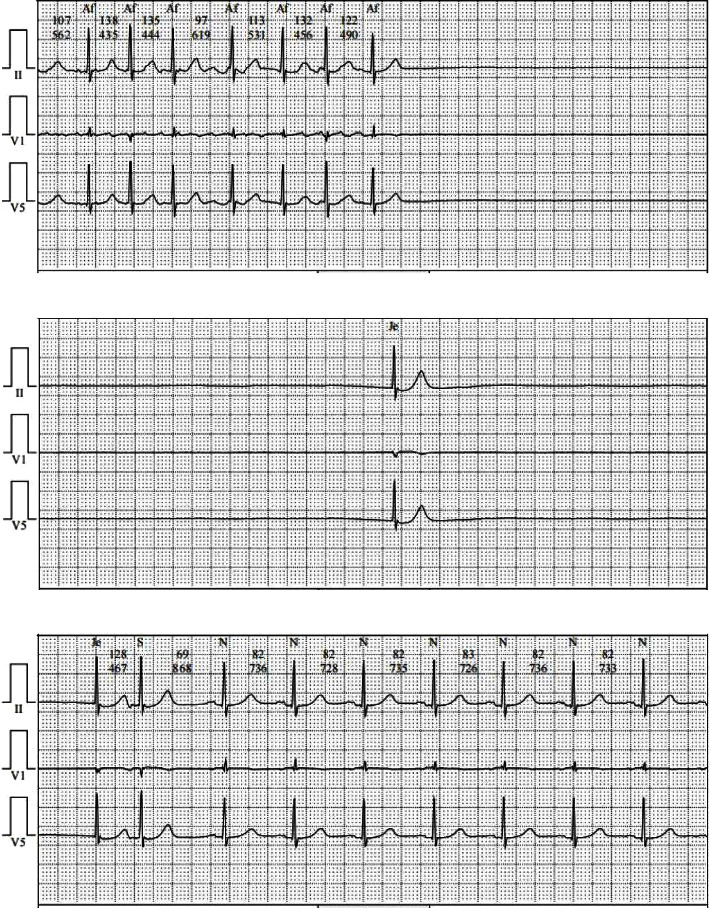
a, b, and c represent a series of continuous cardiac activities monitored by a holter monitor. They exhibit two SAs: the first one lasts for 7.2 seconds, followed by an atrioventricular junctional escape rhythm, and then a second arrest of 3.8 seconds. SA: sinus arrest.

**Figure 2:**
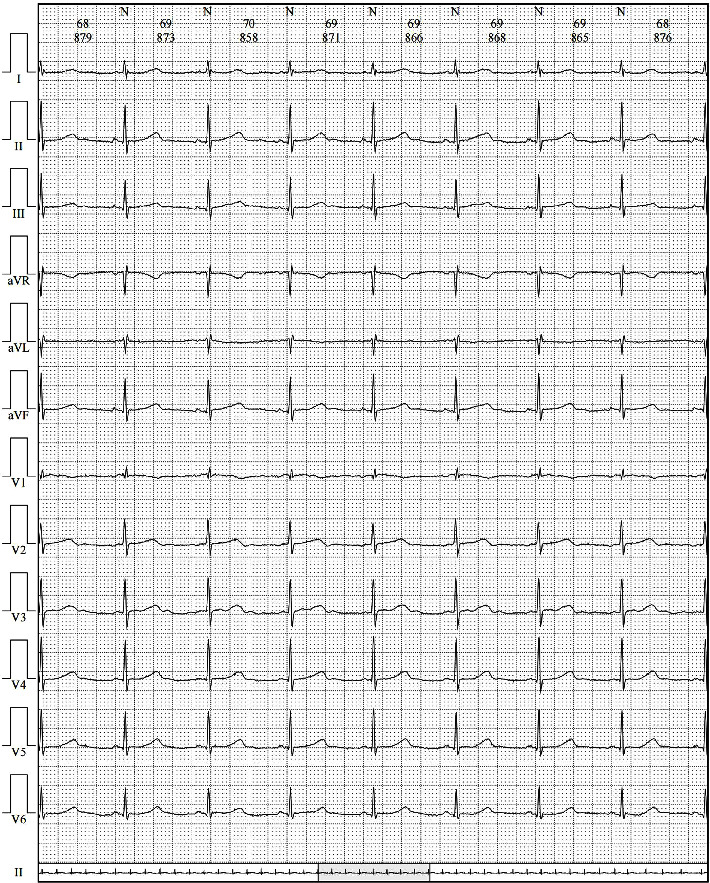
The holter monitor recorded a set of randomly occurring cardiac electrical activity, characterized by sinus rhythm and a heart rate of 69 beats per minute.

The treatment primarily consisted of antiviral therapy using nirmatrelvir/ritonavir and the implementation of mechanical ventilation. The patient tested positive for COVID-19 on the first day of fever and started taking nirmatrelvir (300 mg)/ritonavir (100 mg) on the same day, with a dosage of once every 12 hours for a consecutive period of 5 days. The patient's temperature returned to normal on the 7th day of COVID-19 infection, and subsequent nucleic acid testing yielded negative results, but troponin-I was still high (0.091 ng/mL). On the 16th day of COVID-19 infection (the 11th day after the completion of antiviral medication), the patient's electrocardiogram monitoring revealed frequent episodes of transient cardiac arrest, but the patient's hemodynamic parameters, including blood pressure and oxygen saturation, remained stable without significant changes. The Holter monitor revealed frequent SA, with the longest pause lasting 7.2 seconds (Figure 1). After evaluation by the cardiologist, the patient was deemed to require pacemaker placement; however, the patient's family declined pacing intervention and opted for conservative management. In the subsequent observation, the patient continued to experience recurrent SA, and her troponin-I levels peaked (0.14 ng/mL) on day 18 of COVID-19 infection before beginning to decline. In addition, the frequency of SA gradually decreased about 25 days after COVID-19 infection, and by day 29, electrocardiogram monitoring revealed almost no cardiac arrest, while reexamination of Holter monitor showed no evidence of SA (Figure 2). The SA did not recur during the subsequent 2 months of observation; however, regrettably, the patient remained reliant on mechanical ventilation.

## 3. Discussion

The patient we reported had no history of prior sinus node dysfunction, coronary artery disease, or severe structural heart disease. Following the second COVID-19 infection, there was a progressive increase in the patient's troponin levels and concurrent development of SA, suggesting highly the correlation of the SA to COVID-19 infection. The patient had a medical history of hypertension, diabetes, and atrial fibrillation, all of which exert detrimental effects on the sinus node or cardiac conduction system, potentially leading to the development of bradyarrhythmia [7, 8]. Moreover, there is evidence suggesting that cerebral infarction may also trigger adverse cardiac events [9]. Nevertheless, considering these conditions have been present in the patient for an extended period of time and given that SA occurred shortly after COVID-19 infection onset, we posit that it remains most closely associated with COVID-19 infection.

Ganipisetti et al. [10] reported a case of SA induced by nirmatrelvir/ritonavir administration for COVID-19 infection, with a significant correlation observed between the occurrence of SA and the medication administered. The half-life elimination of nirmatrelvir/ritonavir is approximately 6.05 ± 1.79 hours, with a metabolism rate of 94–97% after 5 half-lives [10]. The patient we report exhibited normal liver and kidney function, suggesting the absence of any metabolic disorders. Therefore, the occurrence of SA on day 11 following drug cessation is unlikely to be attributed to the medication.

It is worth noting that the occurrence of SA in this patient was observed only following the second COVID-19 infection. Research has indicated that immune protection acquired from prior COVID-19 infection diminishes over time [11]. Furthermore, individuals who experience reinfection with COVID-19 are at a heightened risk of mortality and multiorgan sequelae compared to those without reinfection [6]. However, as the strain of SARS-CoV-2 was not tested in both cases of this patient, it is inconclusive whether the virus strain was consistent between the two instances. It should be noted that certain new variants of SARS-CoV-2 possess immune evasion capabilities [12], and different strains may exhibit varying clinical presentations and comorbidities [13, 14].

Currently, there is a growing body of evidence suggesting that the pathogenesis of SA in COVID-19 may be attributed to direct invasion of myocardial cells and cytopathic effects on atrial cells, as well as activation of the immune response [15, 16]. Furthermore, acute complications associated with COVID-19 (e.g., acute pulmonary embolism and severe hypoxemia) and electrolyte imbalances can also contribute to the development of SA [15]. This patient's SARS-CoV-2 nucleic acid test turned negative following treatment; however, she continued to exhibit persistent myocardial damage (troponin continued to rise) and experienced SA. Studies have confirmed that SARS-CoV-2 can persist in the body for an extended period following infection, and even in patients who test negative for nucleic acid after treatment, the virus may continue to replicate within the body [17]. The challenge of viral clearance within the human body may account for this phenomenon we observed.

The case we reported is not unique, as there have been several cases indicating that their patients did not receive any additional therapeutic interventions and experienced a gradual resolution of SA after a period of observation. Babapoor-Farrokhran et al. [18] reported that 2 patients with asymptomatic SA exhibited resolution of the condition after 13 and 14 days, respectively. It is worth noting that the duration of SA in our patient, from onset to disappearance (14 days), was comparable to that observed in these two cases. Ansari et al. [4] demonstrated that the average age of COVID-19 patients with bradyarrhythmia was 56.3 ± 15.2 years old. Although there have been reports of transient SA in patients as young as 83 years old [18], the majority of cases have been observed in younger individuals. The research findings indicate that there is an age-related increase in cardiac collagen content, which is associated with a concurrent rise in fibrosis [19]. This fibrotic process has been observed to be linked to a decrease in the heart rate and a slower sinoatrial conduction time [19]. In addition, the incidence of symptoms and complications resulting from COVID-19 infection has been found to exhibit a positive correlation with age [20]. These findings appear to imply that older patients may face an elevated risk of developing arrhythmias subsequent to contracting COVID-19.

SA is a manifestation of sinus node dysfunction, and it can occur due to various etiologies. In certain situations, sinus bradycardia can be attributed to potentially reversible factors such as acute myocardial infarction, hypothyroidism, medication, infection, and metabolic abnormalities [8]. Reversible SA typically refers to the resolution of recurrent SA through the correction of underlying causes associated with sinus node dysfunction. For those irreversible cases, SA may lead to recurrent symptoms and hemodynamic disturbances in patients, which necessitates further consideration of pharmacological or pacing therapy [8].

In patients with hemodynamically unstable SA, prompt medical therapy and temporary pacing are imperative [8]. Additionally, the identification and elimination of the underlying causes and predisposing causes of arrhythmia are often a crucial step in its treatment. Powell et al. [21] reported that a patient with recurrent syncope was successfully treated for SA following admission for the correction of hypoxemia. However, our patient did not manifest any hemodynamic disturbances. Due to the difficulty in completely eliminating SARS-CoV-2 from the body and limited treatment options for the virus to date, achieving rapid resolution of arrhythmia by addressing its underlying cause may prove challenging.

In patients with hemodynamically stable SA, the consideration of additional pacemaker therapy is warranted in patients with overt symptomatic SA [8]. The efficacy of permanent pacemaker implantation in improving SA in patients with COVID-19 infection has been described in some reports [22–25], while the utilization of temporary pacemakers has also been reported [4, 26]. However, in this case, due to the patient's long-term impairment of consciousness, even if the SA episode presented with symptoms, she was unable to articulate them. In addition, the patient's maximum duration of SA is 7.2 seconds. Given the importance of ensuring patient safety, we maintain that pacemaker placement remains necessary. Indeed, the utilization of temporary pacemakers is also indicated for the management of severe symptomatic SA that can be attributed to a reversible cause [8]. Considering the potential reversibility of SA in relation to COVID-19 infection, opting for temporary pacemaker placement may be more favorable.

The limitation of this case report lies in the absence of strain testing on the patient's two COVID-19 infections. Furthermore, the assessment for SA was not further conducted through electrophysiological studies.

In conclusion, individuals who have recovered from COVID-19 should remain vigilant of the potential for harm caused by reinfection. In addition, the risk of COVID-19-induced arrhythmia, such as SA, should be carefully concerned. Although SA secondary to COVID-19 may be reversible, it is crucial to implement specific treatment measures tailored to the severity of SA.

## Data Availability

The data used to support the findings of this study are available from the corresponding author upon request.
